# Stereotactic body radiation therapy or conventional fractionated radiotherapy combined with immune checkpoint inhibitors? From biological perspectives

**DOI:** 10.3389/fimmu.2025.1578486

**Published:** 2025-08-25

**Authors:** Yanmei Liu, Ganghui Ye, Weijun Chen, Yan Ding, Shenpeng Ying, Shixiu Wu

**Affiliations:** Department of Radiation Oncology, Taizhou Central Hospital (Taizhou University Hospital), Taizhou, China

**Keywords:** SBRT, tumor microenvironment, radiosensitivity, immunotherapy, immune checkpoint inhibitors

## Abstract

We focused on a paper titled “Radiation with immunotherapy may be a double-edged sword—how can we learn from recent negative clinical trials?”, which was published in *JAMA Oncology* recently. Herein, we initially provided three complementary viewpoints from biological perspectives involved in the dynamic alterations of the tumor microenvironment, which may contribute to a more comprehensive understanding of the superiority of stereotactic body radiation therapy (SBRT).

## Background

The PACIFIC-2 trial is a phase III, multi-center, randomized, double-blind clinical trial, which first evaluates the therapeutic efficacy of durvalumab during concurrent chemoradiotherapy (cCRT) followed by consolidation therapy vs. cCRT alone in patients with unresectable stage III non-small cell lung cancer (NSCLC). Regrettably, this trial has not achieved the desired result, distinct from the inspiring outcome of another landmark study, the PACIFIC-1 trial, which has affirmed the clinical efficacy of durvalumab in unresectable stage III NSCLC patients’ maintenance therapy after cCRT. Similarly, to assess the potential benefit from concurrent immune checkpoint inhibitors (ICIs) plus cCRT compared with cCRT alone, multiple clinical trials with negative outcomes have surfaced in recent years, including the CALLA, JAVELIN Head and Neck 100, and Keynote-412 trials. Intriguingly, hypofractionated/stereotactic body radiation therapy (SBRT) plus concurrent ICI therapy exhibited a superior and invigorating therapeutic efficacy in many trials, including the COSINR and PEMBRO-RT trials, which differs from the negative results from concurrent ICIs plus conventional fractionated cCRT.

## The superiority of SBRT

**Figure 1:**
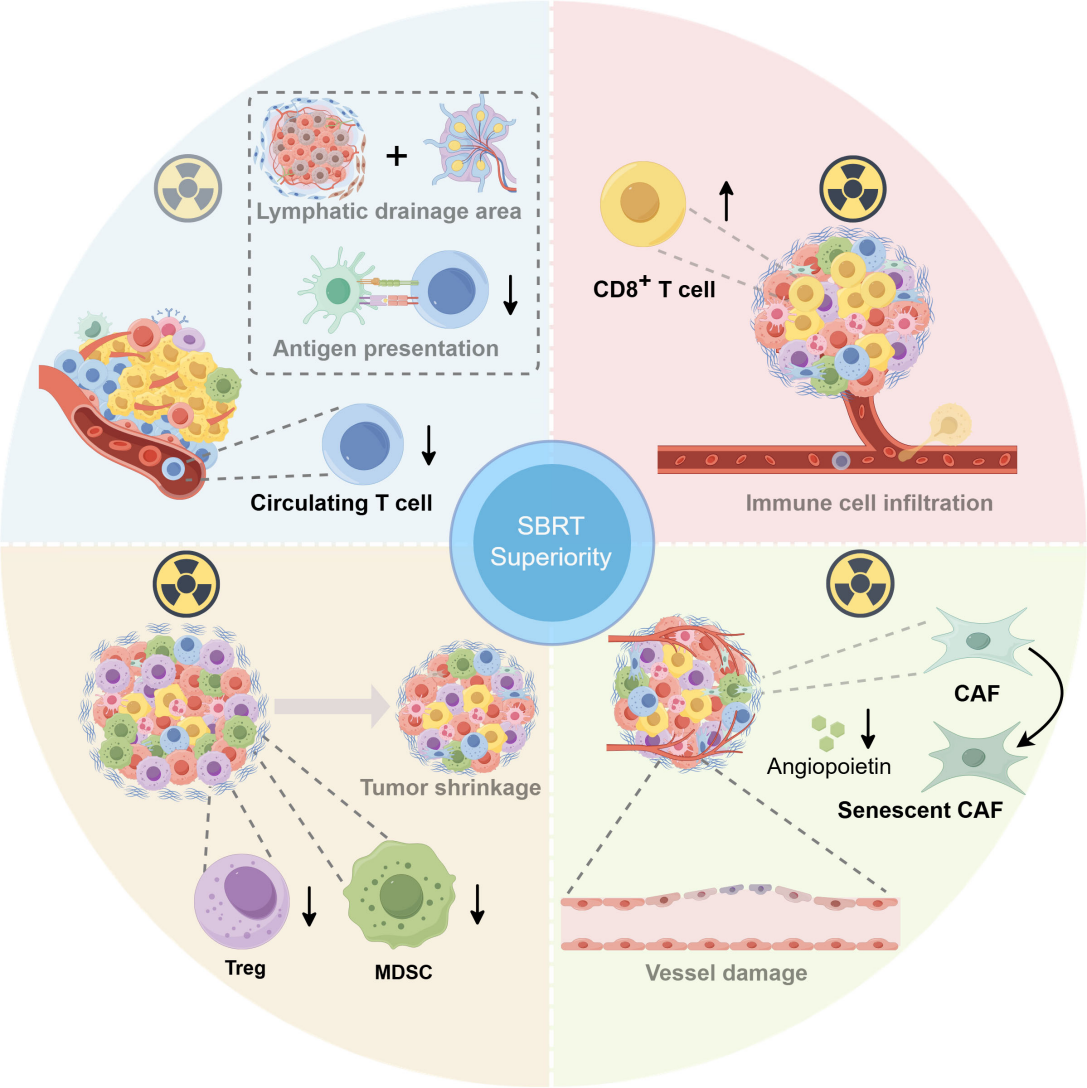
The superiority of stereotactic body radiation therapy (SBRT) on the biological perspective: tumor microenvironment (TME) alteration. Top left: The inhibition of antigen presentation process induced by the irradiation of draining lymph nodes and more severe lymphopenia due to a larger number of fractions is responsible for the failed outcomes of concurrent chemoradiotherapy (cCRT) (1). Top right: Either ablation or hypofractionated radiotherapy (RT) can enhance the CD8^+^ T-cell infiltration. Bottom left: SBRT could indirectly reduce the accumulation of intratumoral immunosuppressive cells [myeloid-derived suppressor cells (MDSCs) and regulatory T cells (Tregs)] by quickly shrinking tumor volume. Bottom right: SBRT could induce severe vascular injury to exacerbate tumor cell death indirectly. Furthermore, the ablation dose of RT can induce cancer-associated myofibroblasts (CAFs) to develop an aging phenotype, significantly reducing angiopoietin secretion while suppressing the function of vascular endothelium. This figure was drawn using Figdraw .

The tumor microenvironment (TME) is a complex ecological ecosystem that tightly correlates with tumor growth, metastasis, and response to anti-tumor therapy. Although ICIs combined with radiotherapy (RT) were considered a promising treatment strategy, not all patients benefited due to the heterogeneity and complexity of the TME. Similarly, extensive research has shown that different radiotherapy regimens may also bring radically varied alterations of the TME and yield different therapeutic effects. In recent years, SBRT has been shown to facilitate the more intense host anti-tumor immune response, increase the incidence of abscopal effect, and enhance the synergistic anti-tumor effects together with ICIs. McGee et al. suggested that the inhibition of the antigen presentation process induced by the irradiation of draining lymph nodes and more severe lymphopenia due to a larger number of fractions is responsible for the failed outcomes of cCRT (1) (**Figure 1**, top left), with the distinct alterations in immune cell groupings identified in the TME following SBRT and conventional cCRT. We believe that exploring the underlying mechanisms for positive results in trials with concurrent ICIs plus SBRT will shed light on optimization using RT and ICIs.

A series of studies have elucidated that either ablation or hypofractionated RT enhanced the CD8^+^ T-cell infiltration and achieved superior therapeutic efficacy combined with ICIs (**Figure 1**, top right). In contrast to conventional fractionated RT (3 Gy, 10 fractions), a single dose of 30 Gy considerably increased CD8^+^ T-cell immune infiltration and resulted in the loss of myeloid-derived suppressor cells (MDSCs), a type of immunosuppressive cell, which significantly increased the tumor response rates and prolonged survival rates in CT26 and MC38 tumor-bearing mice. Interestingly, the addition of a 10-day dose of 3 Gy to the single dose of 30 Gy significantly reduced the CD8^+^ T-cell infiltration and strikingly increased MDSC infiltration, which significantly promoted tumor cell survival and spread (2). Likewise, another study revealed that fractionated RT induced a sustained depletion of infiltrating CD8^+^ T cells in melanoma mouse models. Conversely, single high-dose radiation rendered the intratumoral CD8^+^ T cells to maintain a higher level, which may stimulate stronger anti-tumor immune reactions and correlate highly with lower incidences of distant metastasis and local recurrence (3).

The dysregulation of regulatory T cells (Tregs) and MDSCs exerts negative roles in anti-tumor immune actions. Studies have revealed that high-volume tumors tend to be associated with poor responsiveness to immunotherapy, which may be partially attributed to a rising immunosuppressive cell infiltration, along with a concomitant increase in inhibitory cytokines among multiple tumor types. The incomparable advantage of SBRT vs. conventional fractionated RT in efficiently reducing tumor volume may partially explain its superiority in ICIs’ therapeutic efficacy (**Figure 1**, bottom left). By quickly shrinking single or multiple larger tumors, SBRT indirectly reduced the accumulation of intratumoral immunosuppressive cells and, meanwhile, strengthened anti-tumor immune responses (4). In addition, aiming to uncover the dynamic alterations of peripheral immune cells in patients with prostate cancer treated with three different dose-fractionation strategies, a study indicated that remarkable reductions in peripheral Treg percentage and Treg/CD8^+^ T cell ratio occurred solely at 6 months after the end of SBRT, albeit a progressive increase in peripheral Tregs was noticed during therapy in all three groups. The reduction and functional suppression of peripheral Tregs induced by SBRT may also be correlated with reduced Treg infiltration, contributing to anti-tumor immune activation (5).

Additionally, low irradiation dose (<5 Gy) causes only temporary impairment of vascular function, whereas high irradiation dose (>10 Gy) could trigger sustained tumor vascular damage. In addition to the direct tumor cell killing and promotion of immune killing, SBRT could induce severe vascular injury by triggering endothelial cell dysfunction and apoptosis, indirectly exacerbating tumor killing. In a fibrosarcoma mouse model experiment, a single dose of higher than 15Gy radiation rapidly increased tumor cell death by worsening vascular damage and aggravating tumor hypoxia before anti-tumor immunity activation (6). Furthermore, Hellevik et al. uncovered that a single ablation dose (18 Gy) of radiotherapy induced cancer-associated myofibroblasts (CAFs) to develop an aging phenotype, which significantly reduced their angiopoietin secretion while suppressing the migration ability of vascular endothelial cells, thereby lowering their pro-angiogenic properties (7) (**Figure 1**, bottom right).

## Conclusion

In conclusion, many studies have indicated that a high CD8^+^ T-cell infiltration correlates with a better response to ICIs and improved survival (8). SBRT significantly enhances the CD8^+^ T-cell infiltration and slows its depletion, strengthening the efficacy of anti-tumor immunotherapy. Reciprocally, the stronger CD8^+^ T-cell depletion induced by conventional fractionated RT is detrimental to T cell-mediated immunotherapy, and it is urgent to discover new immune targets. T-cell immunoreceptor with Ig and ITIM domains (TIGIT) was deemed to be associated with T-cell exhaustion. It was shown that the anti-TIGIT antibody therapy decreased Treg infiltration and reversed the CD8^+^ T-cell exhaustion to enhance the anti-PD-L1 efficacy synergistically. Intriguingly, a mouse tumor model experiment indicated that anti-TIGIT antibody exerts significant anti-tumor effects combined with 3 × 8Gy radiation plus anti-PD-L1 treatment only, and no benefit was observed in anti-TIGIT antibody plus 2 × 12Gy radiation plus anti-PD-L1 treatment (9). It triggered us to reflect on whether anti-TIGIT antibodies can overcome the immunosuppression by conventional fractionated RT, subsequently improving ICI efficacy.

Furthermore, with the continuous development of single-cell sequencing and spatial omics technology, there has been an increased awareness of immune cell subsets and their spatial distribution. CD8^+^ T-cell subsets with diverse degrees of exhaustion respond differentially to PD(L)-1 therapy (10, 11). Additionally, the spatial connection between tumor cells and tumor-infiltrating cells (TILs), as well as its dynamic alterations due to immune cell migration, differentiation, and activation, is also closely related to radiotherapy and immunotherapy (12).

Finally, despite the superior therapeutic efficacy of SBRT plus ICI strategy, more clinical trials and translation research are needed for a better understanding of biological mechanisms. Nowadays, many trials (NCT05624996, NCT03795207, and NCT06293690) evaluating the combination of SBRT plus ICIs in various cancer types are ongoing, which may be worth looking forward to.

## Data Availability

The original contributions presented in the study are included in the article/supplementary material. Further inquiries can be directed to the corresponding authors.
